# Preoperative discussion with patients about delirium risk: are we doing enough?

**DOI:** 10.1186/s13741-016-0047-y

**Published:** 2016-09-01

**Authors:** Judith H. Tomlinson, Judith S. L. Partridge

**Affiliations:** 1 Department of Critical Care, University College Hospital, T03, 3RD floor, 235 Euston Road, London, NW1 2BU UK; 2 Proactive care of Older People undergoing Surgery (POPS), Department of Ageing and Health, St Thomas’ Hospital, 9th Floor North Wing, Westminster Bridge Road, London, SE1 7EH UK

**Keywords:** Postoperative complications, Delirium, Preoperative risk assessment, Informed consent, Shared decision-making

## Abstract

Postoperative delirium is a common complication in the older surgical population, occurring in 10–50 % of cases. It is thought to be more common if an individual is identified as frail. Postoperative delirium is associated with poor outcome including higher mortality rates, prolonged length of hospital stay, increased care needs on discharge and longer term post-traumatic stress disorder.

Guidelines from the American Geriatric Society and the National Institute for Health and Care Excellence highlight the importance of risk assessment at the time of the preoperative visit. This enables the perioperative team to plan a care pathway that minimises the risk of delirium occurring postoperatively. Risk assessment also informs a discussion with patient and family regarding their risk, as part of a process of informed patient consent. This is an essential step in conforming to current legal and General Medical Council guidance on the process of consent.

## Commentary

Postoperative delirium (POD) is common in the older surgical population. Variable rates are reported but it is thought to occur in one third of elderly patients having emergency abdominal surgery (Engelberger et al. 2012), up to 50 % of hip fracture patients (Lee et al. 2011) and in 9–11 % of patients when heterogeneous, non-cardiac surgical cohorts are studied (Flinn et al. 2009; Franco et al. 2001). Frail individuals have been found to be at elevated risk (Leung et al. 2011; Jung et al. 2015). Frailty, defined as a state of vulnerability to poor resolution of homeostasis after a stressor event, (Clegg et al. 2013) can be identified preoperatively using validated tools, as advocated by the British Geriatrics Society (British Geriatrics Society 2014).

Delirium is described by the Diagnostic and Statistical Manual of Mental Disorders (DSM) as an acute disturbance in attention, awareness and cognition, which tends to fluctuate over the course of a day and is attributable to an underlying cause (American Psychiatric Association 2013). The patient may display hypoactive, hyperactive or mixed delirium. The pathophysiology of POD is unclear but likely to be multi-factorial in nature. Theories suggest that it may occur as a result of an imbalance in inflammatory responses (Pol et al. 2011; Kazmierski et al. 2013; Kazmierski et al. 2014) or from a deficiency in cholinergic pathways following surgery (Flinn et al. 2009; Cerejeira et al. 2012). The pro-inflammatory cytokine storm that occurs during a surgical procedure is the probable trigger for POD. The anaesthetic is likely to be less responsible although certain drugs used in anaesthesia (such as benzodiazepines and anticholinergic drugs) are known to precipitate or exacerbate POD in vulnerable older patients (American Geriatrics Society 2015).

POD represents a state of acute brain failure and should be recognised as a significant postoperative complication, similar in gravity to an acute kidney injury. POD is independently associated with prolonged length of stay, increased mortality in hospital and at 6 months (Robinson et al. 2009), increased levels of dependence with activities of daily living at discharge and at 6 months (Quinlan and Rudolph 2011), increased likelihood of discharge to an institution (Neufeld et al. 2013; Visser et al. 2015) and with an increased risk of re-admission and re-operation (Large et al. 2013). It has also been associated with a decline in patient reported vitality, physical function and social function after discharge (Abelha et al. 2013) and with the presence of post-traumatic stress disorder at 3 months after surgery (Drews et al. 2015). In medical patients, the combination of frailty and delirium has been shown to confer a particularly poor prognosis (Eeles et al. 2012). Where POD leads to the patient becoming a danger to themselves, physical or pharmacological restraint with resultant deprivation of liberty undertaken in their best interests may be unavoidable, to ensure patient safety (Department of Health 2015). These circumstances can be extremely distressing for patients, family members and staff at the bedside. In addition, POD and its consequences represent a significant financial burden on both health and social care providers (Franco et al. 2001; Markar et al. 2013).

The American Geriatrics Society (AGS) recently published guidelines for the prevention and management of POD in older adults (American Geriatrics Society 2015). The panel of experts highlight the importance of risk assessment in the preoperative setting, aiming to identify the high-risk patient and alter management plans to prevent POD from occurring. The first recommendation from their report states that

Health care professionals caring for surgical patients should perform a preoperative assessment of delirium risk factors, including age greater than 65 years, chronic cognitive decline or dementia, poor vision or hearing, severe illness, and presence of infection.

POD has been found to occur more frequently following emergency surgery, where the speed of the physiological insult is increased and following major surgery, where the extent of the physiological insult is greater. A third important variable is the patient’s preoperative health state (Noimark 2009).

The AGS guideline provides a comprehensive list of patient risk factors (Table 1) and states that if a patient has two or more risk factors, they should be considered at elevated risk of POD, when compared to those with one or no risk factors. This is in keeping with existing risk-prediction models (Marcantonio et al. 1994; Freter et al. 2005). In practice then, those considered at elevated risk of POD are likely to account for a relatively large percentage of the older surgical population.

**Table 1:** Risk factors for postoperative delirium

Risk factors for the development of postoperative delirium	
Age greater than 65 years Cognitive impairment Severe illness or comorbidity burden Hearing or vision impairment Current hip fracture Presence of infection Inadequately controlled pain Depression Alcohol use Sleep deprivation or disturbance Renal insufficiency Anemia Hypoxia or hypercarbia Poor nutrition Dehydration Electrolyte abnormalities (hyper- or hyponatremia) Poor functional status Immobilization or limited mobility Polypharmacy and use of psychotropic medications (benzodiazepines, anticholinergics, antihistamines, antipsychotics) Risk of urinary retention or constipation Presence of urinary catheter Aortic procedures	

Adapted from the AGS guidelines for the prevention and management of postoperative delirium in older adults (Kazmierski et al. 2014)

Assessment of delirium risk is also recommended by the Association of Anaesthetists in Great Britain and Ireland (AAGBI), in its 2014 perioperative care of the elderly guidelines (Griffiths et al. 2014). The working party advocates early recognition and communication of risk throughout the multidisciplinary team. The National Institute of Health and Care Excellence (NICE) issued guidelines in 2010 for the prevention, diagnosis and management of delirium in adult hospital in-patients (National Institute for Health and Care Excellence 2010). Whilst their focus was not specifically on surgical patients, they advised risk assessment at the point of admission to hospital for all patients. Despite the wealth of tools to quantify delirium risk available in the academic literature, the NICE guidelines suggest that any patient who is over 65 years of age, has cognitive impairment, is suffering from severe illness or has a hip fracture is at risk of POD.

National perioperative patient safety reports strongly advocate the use of morbidity and mortality risk assessment tools in the pre-operative setting (National Confidential Enquiry into Patient Outcome and Death 2011; The Royal College of Surgeons of England/Department of Health 2011). Risk assessment serves two important functions. First, the perioperative team can aim to minimise risk by the formation of a careful, evidence-based management strategy and by planning an appropriate level of postoperative nursing care. Second, when a patient is identified as being at high risk of complications, the practitioner should discuss this risk with the patient and their family, as part of a process of informed consent.

A recent Cochrane review concluded that multi-component interventions can prevent delirium in both medical and surgical settings and that monitoring the depth of anaesthesia can reduce the occurrence of delirium following a general anaesthesic. There is currently no clear evidence that other anaesthetic techniques or prophylactic medications are effective in preventing delirium (Siddiqi et al. 2016).

Following the Supreme Court judgement in the case of Montgomery vs. Lanarkshire Health Board in March 2015, the process of consent for medical practitioners has moved away from the Bolam principle. Doctors are required to take “reasonable care to ensure that the patient is aware of any material risks involved in any recommended treatment, and of any reasonable alternatives or varied treatments”(United Kingdom Supreme Court 2015). This is in keeping with existing guidance from the General Medical Council on patient consent, where it is highlighted that all potentially adverse outcomes must be discussed without assumption of which outcome a patient may or may not find important (General Medical Council 2013). Discussion should be individualised and patient-centred and inform shared decision-making (Page 2015).

As an increasingly well-recognised postoperative complication with potentially devastating consequences, POD must be considered an integral part of a preoperative discussion of risk. Aside from being a legal obligation, the discussion may serve to minimise the distress to the patient and their family, caused by such a complication occurring without prior understanding of the nature of the condition (Partridge et al. 2013). The potential for such a discussion to reduce the impact of an episode of POD is an area that requires further research.

Currently, it is unclear whether the responsibility for POD risk assessment and subsequent patient discussion should lie with the surgeon, the anaesthetist or with a physician or geriatrician, where such models of perioperative care exist. In reality, this is likely to vary between centres and will depend to some extent on clinician expertise. Multidisciplinary and cross-specialty team communication is vital to ensure that preoperative delirium risk assessment, risk modification and communication of risk occurs consistently regardless of who undertakes this role. To assist with explaining the potential impact and natural course of an episode of postoperative delirium, the Royal College of Anaesthetists (RCOA) has produced a useful patient information leaflet. It is available online at http://www.rcoa.ac.uk/system/files/PI-Risk7_1.pdf (Cibelli and Smith 2013). The RCOA has outlined its commitment to developing a perioperative medicine curriculum and to promoting the development of perioperative skills and training for anaesthetists, (Royal College of Anaesthetists 2015) working as part of a cross-specialty perioperative medicine team. With increasing numbers of older patients undergoing surgery, it is hoped that a collaborative interdisciplinary and cross-specialty approach will result in improved postoperative outcomes for this high-risk group (National Confidential Enquiry into patient Outcome and Death 2010; The Royal College of Surgeons 2012).

## Conclusions

Postoperative delirium is a common complication in older patients undergoing surgery and is independently associated with poor outcome. Recent guidelines advocate the use of risk assessment to predict the likelihood of occurrence and inform strategies to reduce the frequency of delirium. The perioperative care provider has a responsibility to communicate this risk to patients and carers in the preoperative setting as part of the process of informed consent and shared decision-making.

## Abbreviations

POD: Postoperative delirium
RCOA: Royal College of Anaesthetists
AGS: American Geriatrics Society
